# Interprofessional learning during SARS-CoV-2 (COVID-19) pandemic conditions: the learning project I-reCovEr as a substitute for a rotation on an interprofessional training ward

**DOI:** 10.3205/zma001409

**Published:** 2021-01-28

**Authors:** Sebastian Bode, Alexandra Dürkop, Helena Wilcken, Stephanie Peters, Christine Straub

**Affiliations:** 1 University of Freiburg, Medical Faculty, Medical Center, Department of general pediatrics, adolescent medicine, and neonatology, Center for Pediatrics, Working Group for Teaching and Teaching, Freiburg/Brsg., Germany; 2 University of Freiburg, Medical Faculty, Center for Pediatrics, University Hospital Freiburg, Practical Nurse Training Unit, Nursing Management, Freiburg/Brsg., Germany; 3 University of Freiburg, Medical Faculty, Center for Pediatrics, Practical Nurse Training Unit „Moro“ ward, Freiburg/Brsg., Germany

**Keywords:** interprofessional learning, interprofessional training ward, interprofessional communication, case-based learning

## Abstract

The COVID-19 pandemic has led to massive and aprupt changes in the training of health care professionals. Especially hands-on training can no longer take place in the usual form in everyday clinical practice. Rotations on the interprofessional training ward in Pediatrics (IPAPAED) at the University Medical Center Freiburg, had to be suspended starting March 2020. This report presents the **i**nte**r**prof**e**ssional **Cov**id-19 Replac**e**ment P**r**ogram (I-reCovEr) as an alternative learning format for a rotation on the IPAPAED at the Center for Pediatric and Adolescent Medicine. I-reCovEr offers opportunities for pediatric nursing trainees (n=6) and medical students (n=9) to learn together, taking hygienic and distancing measures into account. Based on a case study, selected learning aspects regarding interprofessional cooperation and communication are targeted. The participants report increased knowledge about the work of the other professional group in the evaluation using the Interprofessional Socialization and Valuing Scale (ISVS) -9A. In comparison to participants of the IPAPAED, however, the self-evaluation did not reveal any self-perceived acquisition of other interprofessional skills or competences. I-reCovEr can therefore serve as an introduction to interprofessional training, but it cannot replace interprofessional learning and working on an interprofessional training ward.

## Zusammenfassung

Die COVID-19 Pandemie hat zu massiven Veränderungen in der Ausbildung der Gesundheitsberufe geführt. Insbesondere sind hiervon die praktischen Ausbildungsanteile betroffen, die nicht mehr in der üblichen Form im klinischen Alltag stattfinden können. Auch die am Universitätsklinikum Freiburg etablierte Interprofessionelle Ausbildungsstation in der Pädiatrie (IPAPÄD) konnte seit März 2020 nicht mehr durchgeführt werden. In diesem Bericht wird das **i**nte**r**prof**e**ssionelle **Cov**id-19 **Er**satzprogramm (I-reCovEr) als alternatives Lernformat für die IPAPÄD am Zentrum für Kinder- und Jugendmedizin Freiburg vorgestellt. In dieser Lehrveranstaltung lernen Auszubildende der Gesundheits- und Kinderkrankenpflege (N=6) und Medizinstudierende (N=9) im Praktischen Jahr gemeinsam unter Beachtung der Hygiene- und Abstandsregeln. Anhand einer Fallvignette werden an vier Terminen ausgewählte Inhalte zur interprofessionellen Zusammenarbeit und Kommunikation erarbeitet. Die Teilnehmenden berichten in der Evaluation mit der Interprofessional Socialization and Valuing Scale (ISVS) -9A von einem Wissenszuwachs über die Arbeit der jeweils anderen Berufsgruppe. Im Vergleich zu Teilnehmenden der IPAPÄD findet sich in der Selbsteinschätzung jedoch kein Erwerb sonstiger interprofessioneller Kompetenzen. I-reCovEr kann daher als Einstieg in die interprofessionelle Ausbildung dienen, das interprofessionelle Lernen und Arbeiten auf einer pädiatrischen Ausbildungsstation jedoch nicht ersetzen.

## Introduction

In 2017, an interprofessional training ward in pediatrics (IPAPAED) was established at the Center for Pediatric and Adolescent Medicine (ZKJ) Freiburg [1]. Here, trainees in pediatric nursing (PN) and medical students (MS) take care of patients in interprofessional teams and learn from, with and about each other. During the two-week rotations on the IPAPAED they are supported by registered pediatric nurses and paediatricians, who received a training in learning assistance [2]. 

Due to the COVID-19 pandemic, in March 2020 the whole study program at the University of Freiburg was suspended and IPAPAED rotations could not be offered. From May 2020, clinical teaching was allowed again in a restricted form, if the recommended hygiene measures, such as appropriate mask wearing, were adhered to. The interprofessional working group on teaching and teaching research at the ZKJ, consisting of nursing practice instructors, social scientists and pediatricians, then developed a program to offer interprofessional (IP) teaching under pandemic conditions. The **i**nte**r**prof**e**ssional **Cov**id-19 Replac**e**ment P**r**ogram (I-reCovEr) was conducted in May and July 2020. Learning objectives include awareness of one's own role and the role of the other professional group, feedback, handover, as well as presentation, diagnosis and therapy of meningitis in infants and performing a lumbar puncture. Selected learning materials from the IPAPAED (see below) were used. Here, the project I-reCovEr and its evaluation are presented and compared to the evaluation of the IPAPAED.

## Project description

**Figure 1:**
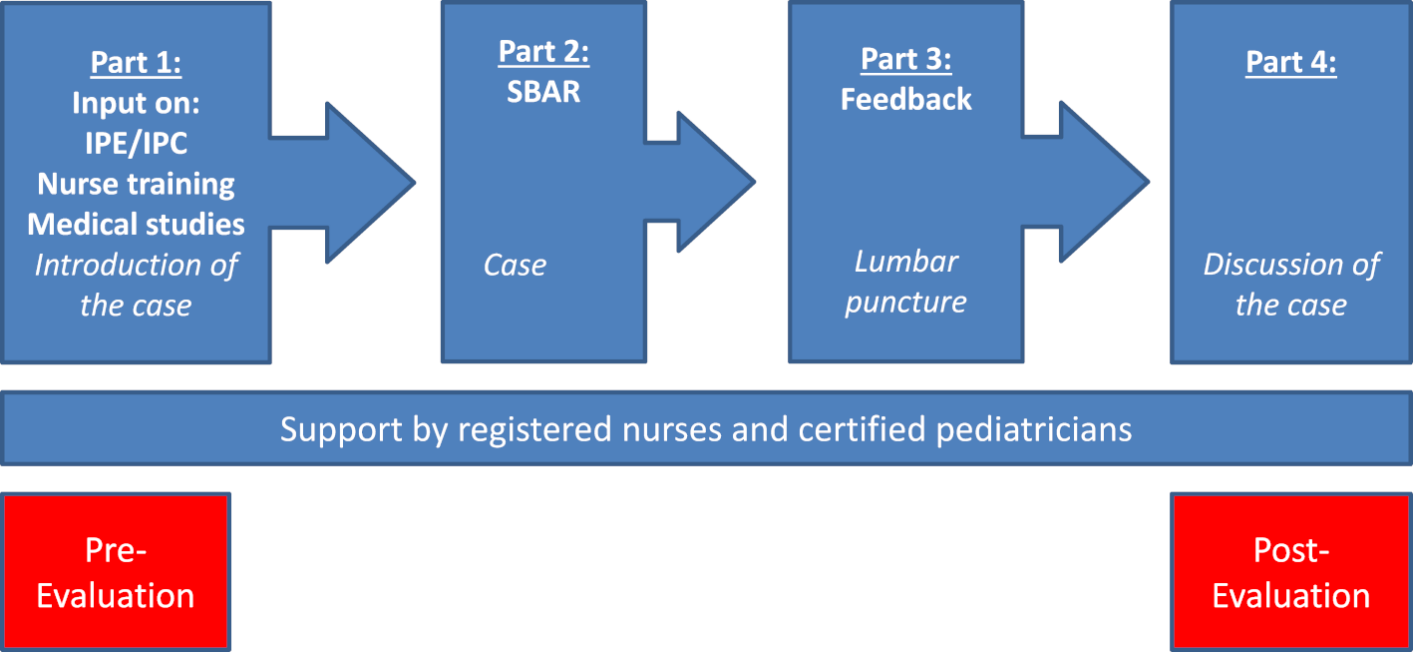
I-reCovEr course outline. Four face-to-face sessions with theoretical input (in bold), interprofessional work on a case study/practical exercise (in italics). IPC = interprofessional collaboration, IPE = interprofessional education.

**Figure 2:**
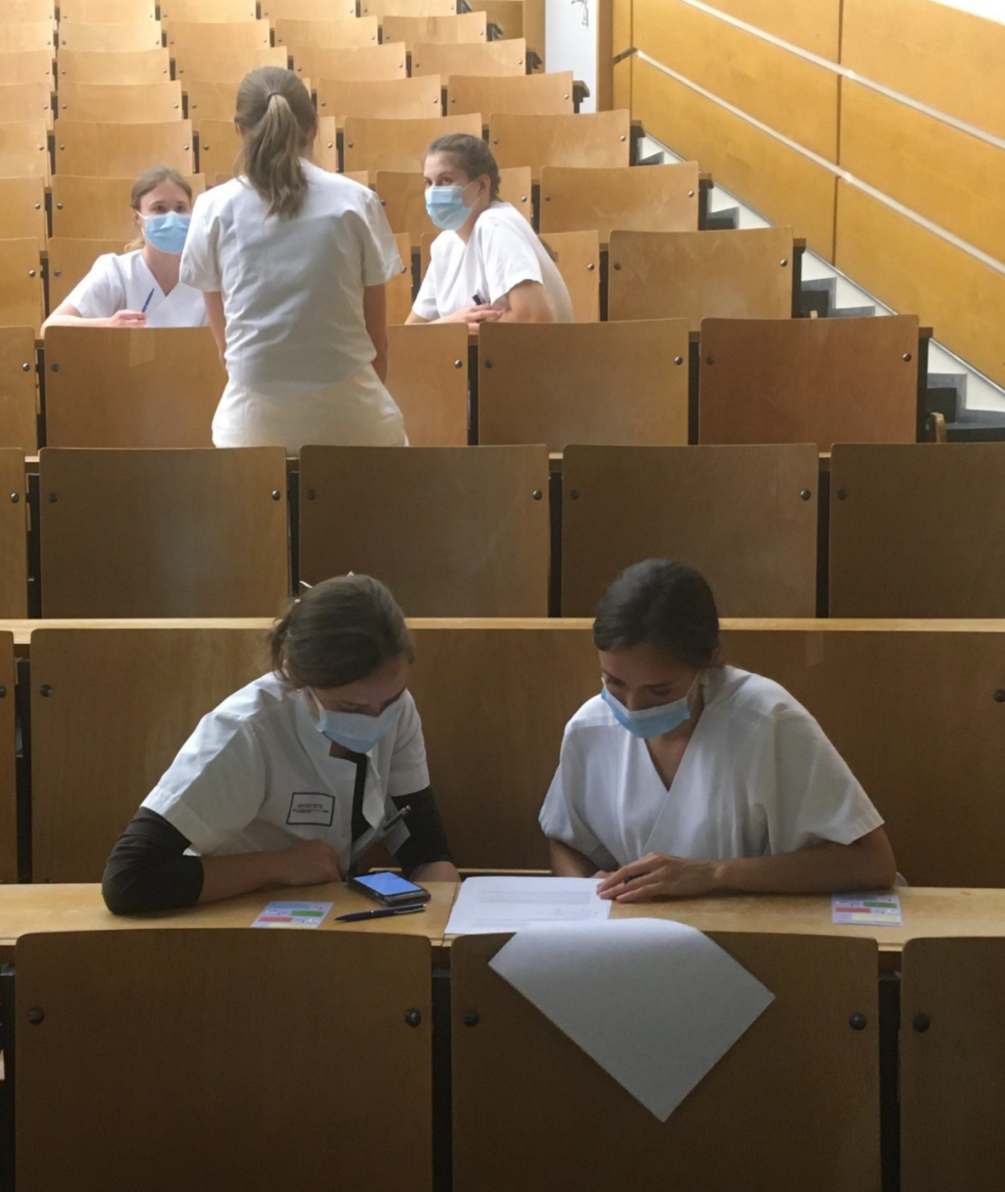
Case work in interprofessional (IP) pairs under pandemic conditions. Registered nurse answers a question of the IP team in the background.

I-reCovEr consists of four 60-minute face-to-face sessions (part 1-4) (see figure 1 (Fig. 1)). The course starts with an introduction to IP learning and IP collaboration. Medical students and nurse trainees present their respective training. Common features of the trainings are discussed. Afterwards, the participants form interprofessional teams to work on a case study while observing the recommended hygiene measures (see figure 2 (Fig. 2)). The case study focuses on the acute presentation of an infant with meningitis. The participants are asked to assess the infant accordingly, discuss aspects of the clinical examination, recommended diagnostics and therapy from a nursing and medical point of view. In the second part, the SBAR (Situation, Background, Assessment, Recommendation) communication tool [3], [4] is presented and applied to the case. Input on feedback and performing a lumbar puncture is given in the third part. The lumbar puncture is prepared by the participants and performed on a dummy. The participants themselves and the IP management team then provide feedback. In the fourth part, the case is completed and discussed. I-reCovEr is evaluated with the ISVS-9A [5] (possible answers from 0=“do not agree at all” to 6=“agree completely”) before and after the course, as are the rotations on the IPAPAED ward. The ISVS-9A asks for attitudes regarding interprofessional cooperation and consists of nine items. In addition, the participants filled out a questionnaire designed for this course with seven items (possible answers from 0=“no knowledge/no significance” to 5=“very much knowledge/very high significance”), which included the free text question "Do you have any further comments on the interprofessional learning unit?".

## Results

Fifteen trainees and students (6 PN and 9 MS) participated in I-reCovEr so far. Twelve participants completed both the evaluation before and after the course. I-reCovEr was evaluated according to German school grades (1=“excellent” to 6=“insufficient”) with m=1.5 (SD±.45). These results are similar to the evaluation results of the IPAPAED (69 TN (m=1.48 (SD±.55)). The participants of I-reCovEr and IPAPAED were comparable in terms of training, gender and age.

Only one item of the ISVS-9A (“I have acquired an increasing awareness of the roles of the other occupational groups in a team”) was rated significantly higher after participation in I-reCovEr than before (pre m=4.33 (SD±.85); post m=5.42 (SD±.64); p<.01). The I-reCovEr questionnaire showed a self-reported increase in knowledge about the other profession (pre m=3.08 (SD±64); post m=4.0 (SD±.58); p<.01). No other significant differences were found. In particular, no self-perceived interprofessional competence acquisition was reported. In contrast, participants of the IPAPAED reported significant knowledge or competence acquisition in 7/9 items of the ISVS-9A and in 5/7 items of the IPAPAED evaluation after a rotation on the IPAPAED.

The free text comments on I-reCovEr praised the opportunity for interprofessional exchange, joint case work and hands-on experience (lumbar puncture).

## Discussion

The project I-reCovEr shows that knowledge about another health care profession can be acquired through joint interprofessional work on case studies. Participants of a rotation on the interprofessional training ward IPAPAED on the other hand rated not only their acquisition of knowledge significantly higher but also reported to have gained interprofessional competencies after their two-week rotation, which could also be shown for other IP training wards [6], [7], [8], [9]. A reason for this might be that at the IPAPAED participants work and learn together every day for two weeks, but in the I-reCovEr course participants only learn together in four face-to-face sessions. In addition, the participants at the IPAPAED take on responsibility for patient care as part of an interprofessional team. The development of corresponding competencies in patient care, assumption of responsibility and team communication cannot be replaced by working with case studies. I-reCovEr can therefore not replace a rotation on an IP training ward. However, the discussion-based learning format of I-reCovEr can be used as an introduction to interprofessional learning or as a minimal IP training format if resources for more complex IP learning formats are not available. In the future, I-reCovEr will focus even more strongly on the acquisition of interprofessional competencies.

The lumbar puncture as an interprofessional learning situation within the framework of I-reCovEr was especially positively emphasized due to its practical relevance. However, appropriate hygienic measures are essential to let participants perform this exercise safely in the context of the COVID-19 pandemic. 

## Conclusion

The interprofessional course I-reCovEr allows for teaching at least some of the learning contents taught on an IP training ward under pandemic conditions. If further IPAPAED courses have to be cancelled, I-reCovEr will be offered again as an alternative, but not as an adequate replacement, for a rotation on the IPAPAED.

## Competing interests

The authors declare that they have no competing interests.
